# Identifying students with dyslexia: exploration of current assessment methods

**DOI:** 10.1007/s11881-024-00313-y

**Published:** 2024-08-29

**Authors:** Johny Daniel, Lauryn Clucas, Hsuan-Hui Wang

**Affiliations:** 1 Durham University, Durham, UK; 2 National Taiwan Normal University, Taipei, Taiwan

**Keywords:** Assessment, Dyslexia, Identification, Reading disabilities

## Abstract

Early identification plays a crucial role in providing timely support to students with learning disabilities, such as dyslexia, in order to overcome their reading difficulties. However, there is significant variability in the methods used for identifying dyslexia. This study aimed to explore and understand the practices of dyslexia identification in the UK. A survey was conducted among 274 dyslexia professionals, including educational psychologists and dyslexia specialists, to investigate the types of assessments they employ, their approach to utilizing assessment data, their decision-making processes, and their conceptualization of dyslexia. Additionally, the study examined whether these professionals held any misconceptions or myths associated with dyslexia. Analysis of the survey data revealed substantial variability in how professionals conceptualize dyslexia, as well as variations in assessment methods. Furthermore, a significant proportion of the survey respondents subscribed to one or more misconceptions regarding dyslexia; the most common misconception identified among professionals was the belief that children with dyslexia read letters in reverse order. The findings highlight the need for standardized approaches to dyslexia identification and debunking prevailing misconceptions. The implications of these findings are discussed, emphasizing the importance of informed policy and practice in supporting students with dyslexia. Recommendations are provided to enhance consistency and accuracy in dyslexia identification, with the aim of facilitating early intervention and support for affected students.

**Supplementary Information:**

The online version contains supplementary material available at 10.1007/s11881-024-00313-y.

Students identified with learning disabilities such as dyslexia are defined as those who demonstrate difficulties in reading skills compared to peers, despite opportunities to learn to read. Intervention efforts to help students overcome their reading challenges generally show greater effects of intervention in early primary grades compared to intervention efforts for students identified in secondary grades (Scammacca et al., 2013, 2016). Indeed, a wealth of data supports early identification as one of the key factors in helping students overcome their reading challenges (see Fletcher et al., 2019).

However, the identification process and the criteria used to identify students with dyslexia have been a subject of ongoing debate (see Elliott & Grigorenko, 2014). While there is consensus in the field regarding what does not constitute dyslexia, there are debates over its specific definition and identification procedures (e.g., Elliott, 2020). Despite the critical importance of accurately identifying dyslexia, there remains a notable gap in the literature regarding the assessment processes used in the UK. Thus, the focus of this study is to investigate what assessments, benchmarks, and procedures assessors such as educational psychologists, dyslexia specialists, and school personnel use to identify school-age children with dyslexia in the UK.

## Dyslexia identification

According the Diagnostic and Statistical Manual of Mental Disorders (DSM-V), dyslexia is defined as “…learning difficulties characterized by problems with accurate or fluent word recognition, poor decoding, and poor spelling abilities…” in the absence of other sensory, emotional, or cognitive disabilities (American Psychiatric Association, 2013, p. 67). Thus, the core observable deficits individuals with dyslexia present are difficulties in decoding and encoding (i.e., spelling) words. In this section, we provide a brief history of dyslexia identification procedures, outline the components that are directly and indirectly associated with dyslexia identification, and highlight some misconceptions that are controversial and may influence diagnostic guidelines and assessment procedures.

Dyslexia identification has a long and complex history. One of the first observations of an individual with dyslexia was made in the late 1800s. In this report, it was noted that a 14-year-old boy who was “bright” and observed to have normal intelligence demonstrated a remarkable inability to read and spell words in isolation (Morgan, 1896). In an attempt to identify the cause of dyslexia, early researchers alluded to theories that this inability to read was associated with some form of “congenital deficits” or “word blindness” or “derangements of visual memory” (Hinshelwood, 1896; Morgan, 1896). It is important to note that these early researchers were vital in raising awareness of conditions associated with the inability to read; however, their inferences were based on observational data and lacked sophisticated methods to support theories associated with cognitive or visual deficits as a cause for dyslexia.

### Models of dyslexia identification

Over the years, researchers have explored different methods to identify students with learning disabilities such as dyslexia. Some of the earlier identification methods relied on hypotheses that visual deficits were a source of dyslexia. For instance, the visual-perceptual deficit model hypotheses (Willows et al., 1993) proposed that reading difficulties are caused by a dysfunction in the magnocellular pathway, which is responsible for processing fast-moving, low-contrast visual information. Based on correlational studies, this pathway was thought to play a crucial role in visual perception, including the ability to perceive letter shapes accurately. However, the causal nature of this pathway has not been established and there is little empirical data to support the visual deficit hypotheses as an explanation for dyslexia (Fletcher et al., 2019; Iovino et al., 1998).

One assessment model which was predominantly used in the last century for identifying students with dyslexia and other learning disabilities, but has been refuted, was the IQ-reading ability discrepancy model. In this identification method, an individual’s assessment scores needed to demonstrate a discrepancy in their IQ test scores and their reading scores. This method aligned with the earliest observations where children were observed to have been “bright” with “normal intelligence” but demonstrated an inability to read. Overwhelming evidence has demonstrated the issues related to the validity of the process and poor reliability in identification (e.g., Fletcher et al., 1998; Francis et al., 2005; Meyer, 2000; Stanovich, 1991; Stuebing et al., 2002). Thus, current evidence does not support the use of this model in the identification process.

More recently, another model of discrepancy known as the patterns of cognitive strengths and weaknesses has been proposed for dyslexia identification (Hale et al., 2014). In this assessment model, individuals’ assessment scores need to demonstrate strengths in certain cognitive domains and weakness in other cognitive domains that are associated with low reading scores (Fenwick et al., 2015). However, multiple studies demonstrate lack of reliability in identification of students with learning disabilities using this assessment method (Fletcher & Miciak, 2017; Kranzler et al., 2016; Maki et al., 2022; Miciak et al., 2015; Stuebing et al., 2012; Taylor et al., 2017). For instance, Maki et al. (2022) observing school psychologists’ dyslexia identification process using the patterns of cognitive strengths and weaknesses model observed that they used considerable amount of time and resources administering cognitive assessments that were associated with low probability of accurate identification.

In addition to the unreliability of this assessment method, another challenge reported is that these assessment procedures are not very informative for educators who have to plan intervention to support students diagnosed with dyslexia (Taylor et al., 2017). For instance, one past meta-analysis reported that interventions that target improvement in students’ cognitive abilities such as working memory have negligible effects on the academic outcomes such as reading (Kearns & Fuchs, 2013).

Another discrepancy model concerns the learning opportunity and poor reading performance (de Jong, 2020), in which learning opportunity is viewed as adequate instruction received by students, and poor reading performance is considered as the unexpected underachievement. In other words, dyslexia is viewed as a discrepancy between reading growth and instructional quality. Based on this perspective, the response to intervention (RTI) model was proposed (Fletcher et al., 2019; D. Fuchs et al., 2012). In the RTI model, all students are screened for reading difficulties, their reading progress is then monitored, and increasingly intense interventions are provided according to their response to progress monitoring assessments (Fletcher & Vaughn, 2009). With this approach, a dyslexia diagnosis can only be fulfilled with severe reading lag and two additional conditions: (a) inadequate growth in reading in general instructional settings and (b) inadequate response to small group or one-on-one evidence-based reading interventions (de Jong, 2020; Fuchs et al., 2012).

The RTI model is supported for substantial advantages, including early intervention and academic prevention, reduction of over-identification, collaboration between general and special education, encouraging evidence-based instruction, providing educational services to students without labeling, and reducing the cost associated with identification process (Fletcher & Vaughn, 2009; D. Fuchs et al., 2012; L. S. Fuchs & Vaughn, 2012). However, the RTI model is not a panacea for dyslexia identification. Issues related to reliability and validity still remain, including problems of identifying adequate instruction and response (Denton, 2012; Kauffman et al., 2011; O’Connor & Sanchez, 2011).

To address the problems of the above-mentioned discrepancy models, one possible solution is to integrate multiple criteria for dyslexia identification. Therefore, hybrid models have been proposed (Fletcher & Vaughn, 2009; Fletcher et al., 2012; Miciak & Fletcher, 2020; Rice & Gilson, 2023). The hybrid models may differ in the assessment implementation (Fletcher et al., 2012) and vary with or without the unexpectedness component (Rice & Gilson, 2023). Current recommendations suggest that a dyslexia diagnosis should be made based on (a) low achievement in reading, (b) inadequate response to evidence-based instructions, and (c) exclusion factors to ensure that low achievement is not due to another disability or contextual factors (Fletcher & Vaughn, 2009; Rice & Gilson, 2023).

Furthermore, assessments are always involved when identifying dyslexia, regardless of which model is applied. It is thus reasonable to consider issues related to the assessments. For example, Miciak et al. (2016) suggested that it is more reliable to incorporate multiple reading assessments and to employ confidence intervals instead of rigid cut-off points during the process of dyslexia identification. In addition to that, culture and language factors should be taken into considerations whenever necessary when administering assessments (American Educational Research Association et al., 2014; Fletcher et al., 2019).

### Distal associations and proximal causes

In this section, we delve into the proximal causes and distal associations of dyslexia, drawing insights from Hulme and Snowling’s (2009) analogy of lung cancer. Emphasizing the significance of reliability and validity in the identification process and its relevance in instructional decision-making within the RTI or hybrid model framework, we aim to explore the key factors that contribute to a reliable identification of students with dyslexia.

Proximal causes. Proximal causes refer to factors that directly and immediately impact the outcome. Taking Hulme and Snowling’s (2009) lung cancer as the exemplar, the gene mutation in the lung tissue would be a direct and proximal cause of lung cancer. Based on this analogy, proximal causes of dyslexia refer to components that directly and immediately produce poor word reading or spelling. Several theoretical models of reading have posited that successful word reading/spelling can be achieved only when multiple proximal causes function together (e.g., Gough & Tunmer, 1986), such as the ability to manipulate sounds or phonological awareness, knowledge of letter-sound relationships or decoding skills, and reading fluency (Gough & Tunmer, 1986; McArthur & Castles, 2017). Failure in any of the above factors could be directly linked to failure in reading or spelling words accurately.

Distal associations. Distal associations refer to factors that have indirect impact on the result. In Hulme and Snowling’s (2009) example, cigarette smoke would be a distal link to lung cancer as it increases the risk of cancer. Regarding dyslexia, distal associations refer to cognitive components that are associated with individuals’ word reading or spelling but are not intrinsic components of reading. In the literature, examples of distal factors associated with reading are working memory, verbal memory, and attention (Burns et al., 2016; Feifer, 2008; McArthur & Castles, 2017).

Although some studies have argued that a comprehensive array of cognitive assessment data, including proximal and distal measures, would contribute to the development of suitable treatment for dyslexia (e.g., Feifer, 2008), other studies have shown that cognitive assessment data is not necessarily helpful for identification and intervention (Burns et al., 2016; Galuschka et al., 2014; McArthur & Castles, 2017). Previous studies have consistently supported the significance of proximal measures for identification and treatment compared to distal measures (Burns et al., 2016; Galuschka et al., 2014). In a meta-analysis that examined the effects of using cognitive data screening and designing interventions among 37 studies, although a small effect was found for distal cognitive measures (i.e., intelligence tests and memory assessments), larger effects were found for proximal measures (i.e., phonological awareness and reading fluency) (Burns et al., 2016). Another meta-analysis has also observed that cognitively focused interventions did not generalize to improvements in reading performance (Kearns & Fuchs, 2013). On the contrary, a proximal intervention, which focuses on the proximal causes of reading, such as phonics instruction and reading fluency training, has shown to be more effective (e.g., Daniel et al., 2021; Scammacca et al., 2016) than a distal intervention that centers on distal associations of reading, such as colored overlays and sensorimotor training (Galuschka et al., 2014).

### Dyslexia misconceptions

The different identification models and evidence supporting or refuting them have given rise to a series of misconceptions that has been reported in mainstream media and academic literature (Elliott & Grigorenko, 2014). Most of these misconceptions stem from procedures that have historical precedence but lack empirical data supporting their use in the identification process. Below we highlight some misconceptions that align with the misconception that having dyslexia is more than deficits in reading and spelling words.

Some portrayals of children with dyslexia note that children see letters and words reversed and this is an indicator of dyslexia. Studies that have explored the letter reversal aspect have compared dyslexic and non-dyslexic individuals and demonstrated that letter reversals are more characteristic of being at a certain stage of reading development, rather than a core aspect of dyslexia; these studies have also reported no significant differences in letter reversals among dyslexic and non-dyslexic children and adults (Cassar et al., 2005; Peter et al., 2020). It is important to note that there is some empirical data to support the hypothesis that individuals with dyslexia misread words due to letter positioning. Some researchers have observed that individuals with dyslexia when reading anagram words (e.g., smile and slime; tried and tired) make migration errors more frequently than control group peers that impact their word reading accuracy and their comprehension (Brunsdon et al., 2006; Friedmann & Rahamim, 2007; Kohnen et al., 2012). In these experiments, individuals with dyslexia might make migration errors wherein they read the word “bowls” as “blows” and this decoding error also impacts their comprehension. However, it is important to highlight that migration errors are different from letter reversals, and we could not locate any studies that observe letter reversals solely in individuals with dyslexia.

Other common misconceptions that are not empirically supported are dyslexic individuals demonstrating high levels of creativity (Erbeli et al., 2021) and sensory-motor difficulties (Kaltner & Jansen, 2014; Savage, 2004). For instance, Erbeli et al. (2021) reviewed 20 studies in their meta-analysis and reported that there was lack of evidence to support the notion of creative benefits for individuals with dyslexia; there were no significant differences in levels of creativity between individuals with and without dyslexia.

There are also misguided recommendations in improving students with dyslexia’s reading skills that align with unsupported theories of visual-perceptual deficit. For instance, there is little evidence to recommend using color overlays (Henderson et al., 2012; Suttle et al., 2018) and specific dyslexic fonts (Galliussi et al., 2020; Kuster et al., 2017; Joseph & Powell, 2022; Wery & Dilberto, 2016) in improving reading skills in students with dyslexia. For example, Galliussi et al. (2020) evaluated the impact of letter form or different fonts on typical and dyslexic individuals’ reading speed and accuracy. Authors reported no additional benefits of reading text in dyslexia friendly fonts compared to common fonts for children with and without dyslexia.

Of concern is that if individuals assessing students for dyslexia adhere to these misconceptions, then this could lead assessors to make erroneous judgments. Thus, in our study, we explore UK dyslexia assessors’ conceptualization of dyslexia and whether they consider these misconceptions as an indicator of dyslexia.

## Literature on dyslexia identification assessment procedures from different countries

In the United States (US), a recent study on identifying school-age students with learning disabilities showed variability in identification criteria, assessments, and diagnostic labels across a wide-range of surveyed educational professionals (Al Dahhan et al., 2021). In a survey of close to 1000 assessors, authors (Al Dahhan et al., 2021) reported assessors using a variety of different criteria when evaluating assessment data and lengthy wait times for individuals to receive assessment and diagnostic results. Similarly, Benson et al. (2020) reported that school psychologists in the US used various identification frameworks, including outdated ones like intelligence-achievement discrepancy. These different frameworks resulted in varied identification decisions, impacting students’ access to support. In Norway, Andresen and Monsrud (2021) found that assessors reported consensus in the types of assessments used to identify students with dyslexia. However, their study also reported that assessors place heavy emphasis on students’ performance on intelligence tests and use reading assessments which lack reliable psychometric properties (Andresen & Monsrud, 2021). A recent systematic review of assessment practices to identify students with dyslexia reported that various dyslexia assessment practices were employed, encompassing cognitive discrepancy and response-to-intervention methods to identify students with dyslexia (Sadusky, et al., 2021). Authors also note that most of the studies reviewed were conducted in the US, with very few studies exploring dyslexia assessment procedures in other countries (Sadusky, et al., 2021). In the United Kingdom (UK), Russell et al. (2012) conducted a case study with one 6-year-old child who was assessed on multiple measures by four different professionals. Authors reported that there was general lack of agreement among professionals on the assessment methodology, which lead to different diagnosis of the child’s areas of needs. However, given this study included only one child, it is hard to generalize these findings to assessment practices in the UK.

These past studies on diagnostic procedures in dyslexia identification highlight the discrepancies in the diagnostic process among assessors leading to inconsistent identification approaches that can impact the services students receive to overcome their learning challenges. To ensure that students with additional needs gain timely access to services, it is essential that all students who have additional needs are identified reliably for support services. More importantly, it is vital to understand that procedures professionals are undertaking to identify students with dyslexia are not only reliable but also valid and align with current recommendations in the field. Furthermore, none of the past studies to our knowledge have explored methods of assessment for students who are English language learners in English-speaking countries, indicating a crucial area for future research to ensure equitable and effective diagnostic practices for this significant student population.

## The UK context: dyslexia identification policy and practice

In the UK, the Equality Act (2010) legally protects individuals with disabilities from discrimination in society, including in educational settings. The Equality Act (2010) provides clarity that it is against the law to discriminate against someone because of “protected characteristics,” one of which is having a disability. “Disabled” is defined as having a physical or mental impairment that has substantial, long-term adverse effects on an individual’s ability to conduct day-to-day activities (Equality Act, 2010). However, neither dyslexia nor specific learning disabilities/difficulties are explicitly mentioned in the Equality Act.

More recently, the Children and Families Act 2014 provides regulations for the Special Educational Needs and Disability Code of Practice (Department of Education, 2014). This regulatory document mentions dyslexia as a condition associated with specific learning difficulties (SpLD). However, it does not provide a definition of what constitutes dyslexia and refers the reader to the Dyslexia-SpLD Trust for guidance. Thus, in the UK, there is no official guidance from policymakers on defining and identifying students with dyslexia or other learning difficulties.

It is also important to state that there are a variety of credentials relating to dyslexia assessment that can be obtained in the UK. For example, the British Dyslexia Association (BDA) offers Associate Membership of the British Dyslexia Association (AMBDA), which is used as an indicator of professional competence in diagnostic assessment. To apply for AMBDA, individuals must have completed an AMBDA accredited Level 7 postgraduate course. These courses are run by various dyslexia organizations, such as Dyslexia Action and Dyslexia Matters, and example courses include a Postgraduate Certificate in Specialist Assessment for Literacy-Related Difficulties and a Level 7 Diploma in Teaching and Assessing Learners with Dyslexia, Specific Learning Differences, and Barriers to Literacy. Completion of one of these courses can then lead to an Assessment Practising Certificate (APC). An APC is used as an indicator that an assessor has competed an AMBDA accredited course and recognizes the knowledge and skills gained from this. This credential is especially important in the UK, as the Department of Education states that a diagnosis of dyslexia will only be accepted as part of a Disabled Students’ Allowance application if it is completed by an assessor holding an APC or if they are a registered psychologist. Because of this, the BDA recommend that all assessors should hold an APC.

## Study purpose

There is currently no clear guidance from policymakers in the UK on the definition and diagnostic procedures of dyslexia. The onus of developing diagnostic procedures and standards relies heavily on various independent professional organizations that develop their criteria for assessments, conduct assessment procedures, and provide diagnostic information to individuals, their caregivers, and school personnel. Apart from one case study that included one participant (Russell et al., 2012), no previous study to our knowledge has explored how independent assessors identify school-age children with dyslexia in the UK. By providing a detailed exploration of the current assessment methods in the UK, this research contributes significantly to the broader understanding of dyslexia identification. We explored the following research questions:

1. How do professional assessors identify students for dyslexia in the UK?
  - What is the common referral process for dyslexia assessment?
  - What types of assessments are used to identify dyslexia?
  - How are standardized measures and cut-off scores utilized in dyslexia diagnosis?
  - How many assessments are conducted and how long does the assessment process take?
  - How do assessors make decisions regarding a dyslexia diagnosis?
  - What assessments are used to assess English language learners for dyslexia?
2. How do professionals conceptualize dyslexia?
3. What is dyslexia assessors’ level of confidence in the validly and reliability of their assessment procedures and their diagnostic judgment?

## Methods

### Ethics

The study received ethical approval from the Ethics Committee at first author’s university. All responses were anonymous and no identifiable information was collected. Participants were able to exit the survey at any time if they no longer wished to participate.

### Recruitment

A recruitment email was sent to various UK-based dyslexia and psychological associations. Four dyslexia associations based in the UK, together with two psychological associations, distributed the survey email and its accompanying link to their members, with the email being sent on one occasion. Also sharing the survey with dyslexia and psychological associations, online searches were conducted to identify potential participants. This involved searching for the terms “dyslexia assessor” and “dyslexia specialist” and specifying the region. The regions included in the search were UK, England, Scotland, Wales, Northern Ireland, North East, North West, South East, and South West of England. These searches allowed us to identify personal websites for individuals offering dyslexia assessment, such as specialist teachers. These individuals were then contacted via the email listed on their website with an invitation to take part in the study and a link to the survey. These professionals were contacted once via email. All survey responses were collected between a 4-week period between January and February 2023.

### Participants

To take part in the survey, participants had to work in a role that involved assessing students for dyslexia, such as a dyslexia specialist, specialist assessor, or educational psychologist. Participants were asked to indicate their current role and qualifications in identifying school-aged students suspected of having dyslexia. See Table 1 for participant demographic information.

**Table 1:** Demographic information

Variable	Category	*N*	%	Total responses
Gender	Male	12	4.38	274
	Female	262	95.62	
Age	20–29	5	1.82	274
	30–39	27	9.84	
	40–49	66	24.09	
	50–59	109	39.78	
	60 +	67	24.45	
Highest degree	High school education	7	2.55	274
	Undergraduate degree	48	17.52	
	Master’s degree	134	48.90	
	PhD/doctorate	27	9.85	
	Other	58	21.17	
Credentials*	Assessment Practising Certificate (APC)	182	79.71	274
	Associate Membership of the British Dyslexia Association (AMBDA) Accredited Course	148	64.82	
	Specific Learning Difficulty (SpLD) Qualification	163	71.39	
	Dyslexia Action MEd in Professional Practice in Dyslexia and Literacy	3	1.31	
	Level 7 Diploma in Teaching and Assessing Learners with Dyslexia, Specific Learning Differences, and Barriers to Literacy	123	53.87	
	Other	68	29.78	
	None	17	7.45	
Current role*	Educational psychologist	35	12.77	274
	Dyslexia specialist	117	51.24	
	Specialist teacher-assessor	178	77.96	
	Special educational needs coordinator (SENCO)	40	17.52	
	Teacher or tutor	74	32.41	
	Disability support worker	3	1.31	
	Lecturer or researcher	4	1.75	
	Other	14	6.13	
Years of experience assessing individuals	Less than 1 year	8	2.92	274
	1–3	35	12.77	
	4–6	55	10.07	
	7–10	27	9.85	
	10 +	149	54.38	
Age group assessed*	Reception or pre-schoolers	32	11.68	274
	Key stage 1 (years 1 and 2)	118	43.07	
	Key stage 2 (years 3 to 6)	208	75.91	
	Key stage 3 (years 7 to 9)	211	77.01	
	Key stage 4 (years 10 and 11)	195	71.17	
	Key stage 5 (years 12 and 13)	169	61.68	
	Higher education and/or adults	126	35.04	

^*^Percentage totals to over 100 as items are not mutually exclusive

### Development of survey instrument

Based on past studies (e.g., Al Dahhan et al., 2021; Andresen & Monsrud, 2021; Benson et al., 2020), we developed a survey to explore how various professionals identify school-age students with dyslexia. The online survey (see Appendix A) included four sections, which were “Demographic Information,” “Assessing and Identifying Students with Dyslexia,” “Conceptualising Dyslexia,” and “Thoughts on the Process of Assessment and Identification.” Before distributing the survey, feedback was obtained from professionals in the field, which resulted in slight changes to the wording of some questions. All survey questions were optional, and participants could choose to skip any of the survey items.

The “Demographic Information” section included nine questions about participants’ background, such as their highest degree and relevant qualifications, their role in identifying students with dyslexia and how long they have worked in this role, and the age groups of students they assess.

The “Assessing and Identifying Students with Dyslexia” section included 25 questions on participants’ assessment and identification process. It included questions about the different types of assessments (e.g., phonological awareness, vocabulary, working memory) they used to identify pupils with dyslexia, the standardized assessments they typically use, their use of benchmarks or cut-off points on these assessments, and their reasons for selecting these assessments. Participants are also asked about the referral process, such as reasons for referral, who generally begins the process, and the average time from referral to diagnosis. The survey also asked participants to report if they assessed individuals who are English language learners and the language of assessments used for this subgroup of individuals.

The “Conceptualising Dyslexia” section had 27 questions that addressed how respondents conceptualize and define dyslexia. The questions focused on the models that participants use to define dyslexia and the criteria they use to identify it. In this section, participants are shown a list of criteria and asked to indicate if they would use these to identify dyslexia. These indicators fell under three subcategories: proximal causes of dyslexia such as poor knowledge of letter names, distal associations of dyslexia such as poor performance on working memory tasks, and myths or misconceptions such as reading letters in reverse order or high levels of creativity.

The “Thoughts on the Process of Assessment and Identification” section had two questions that asked participants about their confidence in their assessment of a student having or not having dyslexia and their perceptions on the reliability of the process in helping them make decisions.

The survey included various types of question items. Many questions allowed respondents to select one or more multiple choice options from a list of choices, for example, questions about the types of assessments used to identify dyslexia or the reasons for referrals (e.g., “What types of assessments do you use to identify students with dyslexia? Choose all that apply.”). Some items used a Likert scale for responses, where participants rate their agreement or frequency of a particular behavior or belief, for example, questions about confidence in assessments (e.g., “How confident do you feel in your assessment of the child as having or not having a reading disability post your assessment? [0 = not confident at all; 10 = certain]”). Participants were also asked open-ended questions to elaborate on their choices such as how they used the assessment data in their diagnostic process.

### Data analysis

We utilized an online polling website for the data collection phase. Upon completion of the data collection process, we downloaded all the collected data onto a spreadsheet. We used the dplyr package (Wickham et al., 2017) in R (R Core Team, 2021) for data cleaning and descriptive analyses.

## Results

### RQ1: how do professional assessors identify students for dyslexia in the UK?

#### What is the common referral process for dyslexia assessment?

Survey participants reported that the most common reason that a parent or school refers a child for assessment is their reading proficiency being below average (62.50% and 59.00%, respectively). Many respondents also reported that parents and school refer a child due to them being unresponsive to classroom reading instruction (65.50% and 35.00%, respectively). However, many are also referred by their parents or school because their cognitive, motor, or visual skills are below average (34.00% and 24.50%, respectively), indicating that more distal indicators are also used to inform referrals. Further reasons for referral provided by participants include students struggling with studies despite showing good general ability, issues with writing and spelling, disparities between verbal and written work, struggling with the curriculum (e.g., working slowly, misreading questions), and running out of time on assessments. Table 2 also shows participants’ responses to the average amount of time it takes from the time they receive a referral to individuals receiving a diagnosis. The majority (59%) of pupils received a diagnosis within 1 month of referral, while 30% received a diagnosis between 1 and 6 months after referral.

**Table 2:** Time from referral to diagnosis

Time frame	Response percentage
Less than a week	5%
More than a week but less than a month	59%
More than a month but less than 6 months	30%
More than 6 months	6%

#### What types of assessments are used to identify dyslexia?

As shown in Table 3, participants were asked to indicate the types of assessments that they use to identify students with dyslexia. Almost all respondents reported assessing reading-related constructs and phonological processing. A vast majority also reported assessing students on various distal measures such as working memory, verbal processing speed, cognitive ability, verbal memory, and reasoning skills. Additionally, Table 4 shows the frequency and types of reading assessments assessors use when conducting assessments with word reading and reading fluency assessments administered most frequently.

**Table 3:** Types of assessments administered for dyslexia diagnosis

Assessment type	Percentage of participants
Reading-related assessments (e.g., reading fluency)	98
Working memory (e.g., letter/number sequencing)	98
Phonological processing (e.g., blending)	97
Writing assessments (e.g., punctuation, story composition)	93
Orthographic processing (e.g., spelling test)	93
Rapid automatized naming (e.g., letters, numbers)	91
Verbal processing speed (e.g., speeded naming test)	90
General cognitive ability (e.g., IQ tests)	89
Oral language assessments (e.g., oral comprehension tests)	82
Verbal memory (e.g., word recall)	79
Parent self-report of current or past learning difficulties/disabilities	76
Reasoning skills (e.g., problem-solving tasks)	69
Visual temporal processing (e.g., response accuracy to visual stimuli)	52
Fine motor skills (e.g., finger isolation, in-hand manipulation)	38
Speech assessments (e.g., expressive language assessments)	31
Auditory processing (e.g., amplitude rise time or intensity discrimination)	22
Other assessments	18

^*^Percentage totals to over 100 as items are not mutually exclusive

**Table 4:** Frequency of reading-related assessments administered during dyslexia assessments

Assessment type	Always	Sometimes	Never	Not reported
Word reading	91.51%	1.89%	0.47%	6.13%
Pseudo word reading	81.13%	10.38%	0.47%	8.02%
Reading fluency	87.74%	5.66%	0.47%	6.13%
Reading comprehension	85.85%	6.60%	0.94%	6.60%

#### How are standardized measures and cut-off scores utilized in dyslexia diagnosis?

To understand participants’ use of standardized measures and cut-off scores, they were asked to report which assessments they use and how they use standardized assessment scores. Across our sample, 80 different standardized assessments were reported as being used during assessments. See Appendix B for a list of the most frequently used standardized assessments. Post assessment administration, a substantial majority (63%) of the participants reported not using cut-off score on standardized assessment to diagnose dyslexia. In contrast, 36% reported utilizing cut-off scores on multiple assessments before completing their diagnostic report. Only one individual in our sample reported using a cut-off score on a single assessment prior to diagnosis.

When asked to explain how they use assessment scores, many reported using the assessments to get an overall picture of a student’s underlying cognitive ability and to look for patterns of strengths and weaknesses that are indicative of dyslexia. It was also often reported that assessors did not use these assessments in isolation, but considered them alongside background information, observations, and reports from parents and teachers. For example, many responses indicated that if a score was low but did not meet a cut-off point, they would consider the assessment scores in relation to background information to determine if, taken together, they indicate dyslexia. Some participants also reported using assessment scores to get a holistic view of strengths and weaknesses and to identify a “spiky” profile in order to build a picture of a student’s areas of need.

#### How many assessments are conducted and how long does the assessment process take?

Participants were asked to report the minimum and maximum number of assessments they use during the identification process and the time the assessment takes. The minimum number of assessments ranged from 1 to 31, with a median of 6, and the maximum ranged from 1 to 50, with a median of 8 assessments.

The minimum assessment time ranged from 45 to 240 min, with a median of 150 min, and the maximum time ranged from 90 to 600 min, with a median of 220 min. These results indicate that there is large variation in the number of assessments used and assessment time, with some professionals, on the extreme end, assessing a child for up to 10 h on up to 50 assessments.

#### How do assessors make decisions regarding a dyslexia diagnosis?

More than four in five respondents make their decisions on a diagnosis independently (85.00%). Of the remaining respondents who work with a team to make decisions, team members included educational psychologists, special education needs coordinators, teachers, other specialists, and families. These results suggest that the vast majority of professionals rely solely on their judgment to make decisions on a child’s diagnosis.

#### What assessments are used to assess English language learners for dyslexia?

Among the 274 survey participants, a subset of 61 respondents indicated that they conduct assessments for individuals who are English language learners. Within the group of 61 assessors who assess English language learners, only a small number, specifically 5, stated that they conduct assessments in the individual’s first language; the remaining 56 reported using the same assessments that are administered to monolingual English-speaking students.

### RQ2: how do professionals conceptualize dyslexia?

#### Familiarity

When presented with the DSM-V definition, which states that dyslexia is characterized by difficulties with reading, spelling, and writing, over two-thirds indicated that the definition was missing elements of cognitive, visual, or motor skills (68.16%). Also, almost a fifth of respondents indicated that the DSM-V definition was inaccurate (19.55%).

#### Dyslexia indicators and myths

Results indicated that almost two-thirds of participants use 5 or more of the proximal indicators (e.g., poor knowledge of letters or letter names, labored or error prone reading fluency) to identify dyslexia (62.15%). Results also demonstrate that 7.91% agree with 5 or more misconceptions as an indicator of dyslexia and close to half of the survey participants associate with at least one misconception as an indicator for dyslexia (43.50%) (e.g., high levels of creativity, use of dyslexia fonts or colored overlays, seeing letters in reverse order). 

#### Models of dyslexia

To understand how participants conceptualize dyslexia, they were asked what constitutes dyslexia. As shown in Table 5, findings indicate that there is large variation in the way that professionals are conceptualizing dyslexia. A large majority reported dyslexia to be a phonological deficit while many also conceptualize dyslexia as a discrepancy between an individual’s reading skills and their cognitive ability (i.e., patterns of strengths and weakness model).

**Table 5:** Assessor conceptualization of what constitutes dyslexia

Dyslexia conceptualization	Frequency (%)
Phonological deficits (deficits in phonological awareness/difficulties in representing speech sounds)	95.48
Patterns of strength and weakness (identify areas of strength and weakness in an individual’s cognitive profile)	82.49
Poor decoders (a group of individuals who struggle with decoding or reading text)	72.88
Intractability to evidence-based reading instruction	60.45
Neurodiverse profile (an individual does not have to experience reading difficulties to be diagnosed with dyslexia and may experience other cognitive difficulties)	46.32
IQ/achievement discrepancy (discrepancy between IQ and reading achievement)	35.59

^*^Percentage totals to over 100 as items are not mutually exclusive

### RQ3: what is dyslexia assessors’ level of confidence in the validly and reliability of their assessment procedures and their diagnostic judgment?

In assessing the confidence levels of dyslexia assessors, the study found that professionals generally felt confident in their diagnostic judgment following an assessment for a child’s potential dyslexia. On a scale from 0 (not confident at all) to 10 (certain), the confidence level was reported with a mean of 8.5, a standard deviation of 1.1, and a median of 9. Similarly, when evaluating the validity and reliability of the assessments they employed in making eligibility decisions, assessors reported high confidence levels, with a mean of 8.3, a standard deviation of 1.3, and a median of 9, on the same confidence scale.

## Discussion

In this study, we explored existing assessment methodologies for identifying school-age children with dyslexia in the UK. We aimed to solicit responses from assessors on their background, their assessment procedures, the types of assessments used, their decision-making process, the types of indicators they use during identification, and their conceptualization of dyslexia. Similar to past studies, there was lack of consensus in the response of assessors on various metrics.

### Validity and reliability of current assessment methods for dyslexia identification

An important takeaway from this study was that most of the survey participants reported that they use reading assessment such as word reading, pseudo word reading, reading fluency, reading comprehension, and spelling in their dyslexia assessment process. These assessment methods align with current recommendations in the field that recommend using academic measures to assess individuals for SpLDs such as dyslexia (e.g., Fletcher et al., 2019). A high percentage of respondents also used some form of writing assessment and/or oral language assessments when evaluating for dyslexia.

Similarly, high percentage of survey respondents also reported using a variety of different cognitive assessments when assessing for dyslexia. Respondents reported administering measures of working memory, general cognitive ability, verbal processing speed, verbal memory, reasoning skills, and visual temporal processing. Given that different assessors used a variety of cognitive assessments, it is important to highlight that this diversity may lead to the identification of varying patterns of strengths and weaknesses in individuals with dyslexia. As a consequence, this lack of consensus in the choice of cognitive assessments employed by assessors raises concerns about the reliability and consistency of the dyslexia identification process.

While past research has demonstrated correlation between cognitive measures and reading assessments, these methods have remained controversial. Little empirical data supports benefits of cognitive assessments in informing intervention efforts. For instance, Stuebing et al. (2002) in their meta-analysis demonstrated that after controlling for pretest reading scores, cognitive measures accounted for 1–2% of explained variance in students’ reading growth. More recently, a pilot study that explored the additional benefits of cognitive training reported no significant benefits of cognitive training on students reading outcomes. In this study (Goodrich et al., 2023), authors assigned preschool children at-risk of reading difficulties to either an early literacy program, early literacy program plus cognitive training, or control. Both early literacy program groups outperformed controls on literacy measures. However, there was no significant differences on literacy outcomes between the literacy only group compared to the literacy plus executive function training group. This study and past reviews consistently highlight little benefits of cognitive training interventions’ effects on academic outcomes (Kearns & Fuchs, 2013). Given this evidence, it is important to question the reason for administering cognitive assessment as they do little to guide intervention efforts to support students’ reading growth.

Another area of discussion is the number of assessments assessors use to identify students for dyslexia. A general recommendation in the field is to use more than one assessment for identification, as a single measure may underrepresent a construct (Fletcher et al., 2019). The median number of minimum assessments reported by assessors was six, and the median maximum number of assessments reported was eight. While this indicates a multi-faceted approach, the fact that almost 2/3rd of the sample reported not using cut-off scores raises questions about how diagnostic decisions are made. While the avoidance of strict cut-off scores aligns with the understanding that word reading abilities exist on a continuum, the lack of their use raises questions about how assessors are synthesizing the results of multiple assessments to determine a diagnosis. Confidence intervals, which account for measurement error and provide a range of plausible values, offer a more accurate and inclusive approach to identifying reading difficulties (Miciak et al., 2016) and could potentially address this ambiguity. Thus, it was perplexing to see that most assessors were not making normative comparisons to guide their decision-making. Another challenge is that almost all assessors use a blend of academic (e.g., reading) and cognitive assessments (e.g., working memory) to identify strengths and weaknesses or to identify a “spiky” profile. Past research on evaluating patterns of strengths and weakness has demonstrated this process to be unreliable and lacking validity (Fletcher & Miciak, 2017; Maki et al., 2022).

There are no guidelines from policymakers in the UK to the holistic process of evaluating students’ assessment scores, raising concerns about the reliability of this process. This concern is supported by one past case study in the UK, which found that different professionals came to very different conclusions of a child’s areas of academic needs based on their evaluation of the assessment data (Russell et al., 2012). Thus, the question is would different assessors come to different conclusions based on their own holistic evaluation of assessment data?

Our findings related to the variability in diagnostic procedures and conceptualization of dyslexia suggest a need for government policy to guide the assessment procedures for students with dyslexia. For example, in the United States, the Individuals with Disabilities Act (IDEA, US Department of Education, 2006) clearly states that “The Department does not believe that an assessment of psychological or cognitive processing should be required in determining whether a child has an SpLD. There is no current evidence that such assessments are necessary or sufficient for identifying SpLD. Further, in many cases, these assessments have not been used to make appropriate intervention decisions” (p. 46,651). Similar guidance is needed for more reliable identification processes in the UK.

Another important area to highlight is that one past study in the UK has reported parental income to be a significant predictor of a child being diagnosed with dyslexia; the likelihood of being identified as dyslexic increases with higher income (Knight & Crick, 2021). For parents in the UK, assessing their child for dyslexia could cost anywhere between £500 and £700. This raises questions of equity and who can afford these assessments as 60% of households in the UK earn less than £799 per week (Office of National Statistics, 2023). Given the high costs of assessments and the post-pandemic cost of living crisis in the UK, we wonder how many households have disposable incomes to afford paying for dyslexia assessments. We wonder if there is a need for cognitive assessments and, if not, would reducing the number of assessments help assessment institutions to reduce the cost of assessments to make it more equitable and accessible to the general public. It is important to note that the National Health Services in the UK does not cover the cost of dyslexia assessments and this cost has to be incurred by caregivers.

### Assessor conceptualization of dyslexia

All survey participants (100%) reported that they are “very familiar” with dyslexia. However, it was perplexing to observe that only small proportion of our sample reported agreeing with DSM-V definition of dyslexia that defines dyslexia as issues with word reading, reading fluency, and spelling words. When probed further on how assessors conceptualize dyslexia, majority reported it being a phonological deficit, inadequate decoding skills, and lack of response to evidence-based reading instruction. However, a substantial proportion of the sample also aligned with dyslexia being conceptualized as patterns of strengths and weaknesses or a discrepancy between IQ and achievement. Our data suggests that although a resounding number of study participants align with the DSM-V definition of dyslexia, they also have a strong commitment to cognitive assessments as an integral aspect of identification. This lack of consensus is consistent with past research on the lack of consensus among what constitutes dyslexia (e.g., Al Dahhan et al., 2021; Ryder & Norwich, 2019; Sadusky et al., 2021).

Additionally, we also wanted to explore if dyslexia assessors subscribe to myths or misconceptions about dyslexia. The common misconceptions that dyslexia assessors reported as being an “indicator of dyslexia” were that individuals with dyslexia read letters in reverse orders (61%), they see letters jumping around (33%), they have high levels of creativity (17%), they report motor skills issues or clumsiness (17%), and they struggle to read words only when text is displayed in certain colors (15%) or fonts (12%). This suggests that there are many assessors that align with misconceptions to inform their decisions surrounding dyslexia diagnosis. Empirical data does not support these to be indicators of dyslexia (e.g., Henderson et al., 2012; Kuster et al., 2017). Thus, there is a need for dyslexia and psychological associations in the UK to ensure that these misconceptions are directly addressed in their certification modules. This is especially important as a majority of respondents reported using the data holistically to evaluate their diagnosis procedure and these misconceptions could influence assessors’ judgments and could potentially be associated with identification errors.

### Assessor confidence

We observed that assessors generally reported high levels of confidence in the validity and reliability of the diagnostic process and their diagnosis. This is consistent with previous findings in both educational (Maki et al., 2022) and clinical settings (Al Dahhan et al., 2021), where practitioners generally reported high confidence in their ability to identify students with specific learning disabilities/difficulties, especially those assessors who had received more training. However, this reported confidence contrasts with the concerns raised in the present study about the reliability and validity of methods employed (such as the patterns of strengths and weakness), the pervasive use of a variety of cognitive assessments, the lack of framework on how assessment data is to be used for diagnosis, and the belief in dyslexia misconceptions that a large proportion of the sample subscribes to. This discrepancy, echoing Maki et al.’s (2022) findings of a potential disconnect between accuracy and confidence, suggests that decision-making confidence might be misplaced if it is not underpinned by standardized and widely accepted identification methods. Hence, while assessors are confident in their diagnostic capabilities, this confidence may be problematic if the identification methods themselves are flawed or inconsistently applied. Further research exploring the relationship between training, experience, and diagnostic accuracy in this context is warranted.

### The English language learner dilemma

There is little data in the research literature to shed light on dyslexia assessment practices for English language learners. In our survey, we asked UK dyslexia assessors if they assessed individuals who were English language learners. Approximately 30% of our sample reported assessing English language learners for dyslexia. Within this subsample, a majority (92%) reported that they did not assess English language learners in their first language and generally used the same assessments they used for monolingual English speakers. This is an area of concern as assessing individuals on assessments that are in their second language may impact the validity of assessors’ interpretation of assessment data.

While past researchers (Fletcher et al., 2019) recommend selecting assessments that are linguistically and culturally sensitive to make accurate inferences, there may be practical challenges. For instance, some respondents reported that they have been unable to access assessments in students’ first language, despite asking their local authority for support in doing so. This indicates assessors’ willingness to assess individuals in culturally and linguistically sensitive assessments, but the lack of available resources may be a potential barrier. Thus, improving assessors’ knowledge and access to assessments in students’ first language may be one step towards administering culturally and linguistically fair assessments that can lead to improved identification decisions for this subpopulation of individuals.

### Limitations

A notable limitation of this study is that we are not aware of the survey response rate. Although post code data shows that our sample was recruited from all over the UK, it is not certain that this sample’s assessment practices are representative of all UK dyslexia assessors. Another limitation is that survey questions were limited to dyslexia identification and did not elicit responses on identification of other learning disabilities/difficulties such as reading comprehension difficulties, math difficulties, and/or writing difficulties.

### Future recommendations and conclusion

Our study demonstrates that there is a general lack of consensus among assessors on the process of dyslexia identification. While many subscribe to the notion of dyslexia being a deficit in core areas of reading, several others subscribe to dyslexia being a discrepancy between individuals’ reading and cognitive profiles. There is a clear need in the UK for policymakers to clearly define dyslexia and provide assessment guidelines. Nationally defined identification pathways would be useful in providing guidance to various assessment institutions and this alignment could lead to a cohesive model for reliable identification of learning difficulties such as dyslexia.

## Supplementary Information

Below is the link to the electronic supplementary material.

ESM 1 (DOCX 26.9 KB)

## Data Availability

The data that support the findings of this study are available in the UK Data Service ReShare repository. The data have been stored in accordance with institutional guidelines and are accessible for replication purposes. For further inquiries, please contact the corresponding author at johny.r.daniel@durham.ac.uk.
