# Supplementary material for: Identifying students with dyslexia: exploration of current assessment methods
Source: Ann Dyslexia. 2024 Aug 29;75(1):19–41. doi: 10.1007/s11881-024-00313-y (PMC11954703; doi:10.1007/s11881-024-00313-y)
Supplement: Supplementary file 1 — (DOCX 26.9 KB) [file 11881_2024_313_MOESM1_ESM.docx]

**Appendix A**

Survey Instrument

**Survey Purpose**
This exploratory study has been funded by the British Educational Research Association to explore the topic of Learning for All. Our perspective is that for learning to occur for all students, we first need to identify students who have additional needs to ensure they receive the services that can support their learning. Thus, with this aim, the purpose of this survey is to understand how various professionals identify students with dyslexia in the United Kingdom. 

Your responses to this questionnaire are entirely voluntary and will be used, anonymously, in our research. You may withdraw your participation at any time. By completing this questionnaire, you agree to participate in our study. 

For our part, we agree to report the findings only as aggregate data. A checkbox indicates that you have read and understood the terms of this agreement, agree to participate in this study, and allow the use of your information in the questionnaire for research purposes. 

This study has received ethical approval from Durham University's Ethics Committee. 

For any questions or queries about the survey, please email - johny.r.daniel@durham.ac.uk

___ Yes, I agree

___ No, I don’t agree

**Section 1: Demographic Information**

Please tell us a little about yourself

Gender

- Male
- Female
- Nonbinary

Age

_______ (in years)

What are the first two letters of your work postcode?

______ (E.g., If the post code is **DH**1 3LE, enter **DH**)

What is your highest degree?

- GCSEs/O-Levels (left school at 16)
- A-Levels (left school at 18)
- Tertiary vocational qualification
- An undergraduate degree
- A masters degree
- PhD/Doctorate
- Other (please specify) ______

Do you have any other credentials that you need to work with students with dyslexia and other learning difficulties?

- Assessment Practising Certificate (APC)
- Associate Membership of the British Dyslexia Association (AMBDA) Accredited Course
- Specific Learning Difficulty (SpLD) Qualification
- Dyslexia Action MEd in Professional Practice in Dyslexia and Literacy
- Level 7 Diploma in Teaching and Assessing Learners with Dyslexia, Specific Learning Differences, and Barriers to Literacy
- Other (please specify) ________
- I do not have any other credentials

Does your current/past role involve assessing students for dyslexia or other learning difficulties?

__ Yes

___No

What is your current role in identifying students with dyslexia or other learning difficulties? Select all options that apply to you.

- Educational psychologist
- Practitioner psychologist
- Dyslexia specialist
- Specialist teacher-assessor
- Special Educational Needs Coordinator (SENCO)
- Paediatrician or medical professional
- Teacher or tutors
- Disability support worker
- Lecturer or researcher
- I do not assess or I am not part of an assessment team that identifies students with learning difficulties such as dyslexia (END SURVEY)
- Other _________

How many years of experience do you have in the above selected role?

Less than one year

- 1 – 3
- 4 – 6
- 7 – 10
- 10+

What age group of individuals do you work with to assess them for dyslexia or other learning difficulties? (Select all options that apply to you)

- Reception or pre-schoolers
- KS1 (Years 1 and 2)
- KS2 (Years 3 and 6)
- KS3 (Years 7 and 9)
- KS4 (Years 10 and 11)
- KS5 (Years 12 and 13)
- Higher education and/or Adults

**Section 2: Assessing and identifying students with dyslexia**

Are you familiar with the term dyslexia?

- Extremely familiar
- somewhat familiar
- not at all familiar

Do you make decisions on students’ diagnosis of dyslexia

- Independently
- As part of a team
  - Who is part of team? Please list professionals you work with as a team (e.g., SENCO, educational psychologist) _________________________

Approximately, how long does it take from once you receive a referral to the child receiving their diagnosis

- Less than one week
- More than a week but less than a month
- More than a month but less than six months
- More than six months but less than a year
- More than a year

What are some of the reasons that children get referred to you for assessments of dyslexia? Choose all that apply.

- Parent reports child's reading proficiency as below similar age peers
- Parent reports child has not responded to effective reading instruction
- Parent reports child's cognitive, visual, or motor skills as below similar age peers
- School personnel reports child's reading proficiency as below similar age peers
- School personnel reports child has not responded to effective reading instruction
- School personnel reports child's cognitive, visual, or motor skills as below similar age peers
- Other (please specify) ________

What types of assessments do you use to identify students with dyslexia? Choose all that apply.

- Reading assessments (e.g. reading fluency tests, word reading, reading comprehension)
- Oral language assessments (e.g., oral comprehension tests, vocabulary tests)
- Writing assessments (e.g., punctuation, story composition)
- Phonological processing (e.g., blending)
- Orthographic processing (e.g., spelling test, orthographic choice)
- Rapid automatized naming
- General cognitive ability (e.g., IQ tests)
- Visual temporal processing (e.g., response time and response accuracy to visual stimuli)
- Working Memory (e.g., forward and backward digit span, letter/number sequencing)
- Verbal memory (e.g., word recall)
- Verbal processing speed (e.g., speed naming test)
- Fine motor skills (e.g., finger isolation, in-hand manipulation)
- Speech assessments (e.g., expressive language assessment)
- Auditory processing (e.g., amplitude rise time or intensity discrimination, auditory reaction time)
- Reasoning skills (e.g., problem solving tasks)
- Parental self-report of current or past learning difficulties
- Others (please specify)

Which of the following measures of reading do you use to assess students with dyslexia, and how frequently do you use them?

- Word Reading (Never, Sometimes, Always)
- Pseudo-word reading (Never, Sometimes, Always)
- Reading fluency (Never, Sometimes, Always)
- Reading Comprehension (Never, Sometimes, Always)
- Other ________

Which standardised assessments do you use?

- Woodcock-Johnson IV Tests of Cognitive Abilities (WJIV Cog)
- Test of Auditory Processing Skills (TAPS-4)
- Wechsler Individual Achievement Test (WIAT III UK)
- Wide Range Achievement Test (WRAT5)
- Feifer Assessment of Mathematics (FAM)
- Adult Reading Test (ART-2)
- Feifer Assessment of Reading (FAR)
- Kaufman Test of Educational Achievement (KTEA-3)
- Test of Memory and Learning (TOMAL 2)
- Wechsler Abbreviated Scale of Intelligence (WASI-II)
- Woodcock-Johnson IV Tests of Achievement (WJIVAch)
- Woodcock-Reading Mastery Tests (WRMT-III)
- Other (please specify)

After completing the assessments, what criteria do you use toidentify those with and without dyslexia?

- Score cut-off on one assessment
- Score cut-off on multiple assessments
- I do not use cut-off scores

Please explain how you use assessment scores ________________________________

How many total different assessments do you use in your diagnostic process of dyslexia?

Minimum number of assessments: _______

Maximum number of assessments: _______

What is the minimum and maximum assessment time when assessing a student for dyslexia?

Minimum time (minutes): _______

Maximum time (minutes): _______

Do you assess individuals who are considered English language learners for dyslexia?

___ Yes

___ No

If yes, do you assess individual who are English language learners

- With assessments in their first language? Yes/No
- With the same assessments listed above that you use for monolingual English speakers? Yes / No

If you use assessments in their first language, please list the assessments you use: _________________________

**Section 3: Conceptualising Dyslexia**

"Dyslexia is a term that refers to difficulty in acquiring and processing language that is typically manifested by the lack of proficiency in reading, spelling, and writing. People with dyslexia have difficulty connecting letters they see on a page with the sounds they make. As a result, reading becomes slow and effortful and is not a fluent process for them." (DSM-V)

This definition of dyslexia

- Is accurate
- Is accurate but is missing elements of cognitive/visual/motor skills
- Is inaccurate

Which of the following models do you use to conceptualise what constitutes dyslexia? Choose all that apply.

- Phonological deficits (deficits in phonological awareness/difficulties in representing speech sounds)
- Poor decoders (a dyslexic subgroup of a larger group of people who struggle with decoding text)
- Intractability to evidence-based reading instruction (interventions are ineffective for the individual)
- Neurodiverse profile (an individual does not have to experience reading difficulties to be diagnosed with dyslexia, and may experience other cognitive difficulties)
- IQ/achievement (discrepancy between IQ and reading achievement)
- Patterns of Strength and Weakness (identify areas of strength and weakness in an individual's cognitive profile)
- Other (please tell us more)

Which of the below do you:

- think is an indicator of dyslexia?

- never use as a criterion to diagnose dyslexia?

- Poor knowledge of letters in the alphabet
- Poor awareness of sounds in the alphabet(phonological awareness)
- Poor performance on non-word reading tests(e.g., um, hap)
- Poor performance on memory tests
- Poor knowledge of letter names
- Poor performance on reading comprehension tests
- Poor performance on reading fluency tests
- Poor performance on spelling tests
- Poor performance on listening comprehension tests
- Child reports seeing letters jumping around on the page
- Late language development
- Sensory-motor difficulties and/or clumsiness
- Poor performance on writing tests
- Demonstrates poor reading skills only when text is displayed in specific fonts
- Demonstrates poor reading skills only when text is displayed in certain colour backgrounds
- Low motivation to read
- Child lives in poverty or is from a low socio-economic background
- Poor performance on IQ tests
- Reading letters in a reverse order
- Poor visual perception
- Hyperactivity
- High levels of creativity
- Family history of dyslexia or other learning difficulties

**Section 4: Your thoughts on the process of dyslexia assessment and indentification**

How confident do you feel in your assessment of the child as having or not having a reading disability post your assessment?

(0 = Not confident at all; 10 = Certain)

How confident do you feel that the assessments you are using are valid and reliable in helping you make eligibility decisions?

(0 = Not confident at all; 10 = Certain)

**Appendix B**

Most Frequently Reported Standardized Measures Administered

| Standardized Measure | Frequency Percentage |
| --- | --- |
| Test of Memory and Learning (TOMAL-2, Reynolds & Voress, 2007) | 79 |
| Wechsler Individual Achievement Test (WIAT-III-UK, Wechsler, 2017) | 55 |
| Wide Range Achievement Test (WRAT-5, Wilkinson & Robertson, 2017) | 55 |
| Adult Reading Test (ART-2) | 36 |
| Comprehensive Test of Phonological Processing (CTOPP-2, Wagner et al., 2013) | 63 |
| Test of Word Reading Efficiency (TOWRE, Torgesen et al., 2012) | 59 |
| Detailed Assessment of Speed of Handwriting (DASH, Barnett et al., 2007) | 47 |
| Wide Range Intelligence Test (WRIT, Glutting et al., 2000) | 41 |
